# Serum Levels of Irisin Predict Cumulative Clinical Outcomes in Heart Failure Patients With Type 2 Diabetes Mellitus

**DOI:** 10.3389/fphys.2022.922775

**Published:** 2022-05-16

**Authors:** Alexander A. Berezin, Michael Lichtenauer, Elke Boxhammer, Ivan M. Fushtey, Alexander E. Berezin

**Affiliations:** ^1^ Zaporozhye Medical Academy of Postgraduate Education, Zaporozhye, Ukraine; ^2^ Department of Internal Medicine IIDivision of Cardiology, Paracelsus Medical University Salzburg, Salzburg, Austria; ^3^ Division of Cardiology, Department of Internal Medicine II, Paracelsus Medical University Salzburg, Salzburg, Austria; ^4^ Department of Therapy and Endocrinology, Zaporozhye Medical Academy of Postgraduate Education, Zaporozhye, Ukraine; ^5^ Internal Medicine Department, State Medical University of Zaporozhye, Zaporozhye, Ukraine

**Keywords:** heart failure, irisin, natriuretic peptides, type 2 diabetes mellitus, prediction

## Abstract

**Background:** The aim of this study was to investigate the role of serum irisin level in predicting clinical outcome in heart failure (HF) patients with type 2 diabetes mellitus (T2DM).

**Methods:** 153 T2DM patients with HF aged 41–62 years were prospectively recruited for the study. Serum levels of irisin and NT-proBNP were measured by ELISA. Laboratory tests including HbA1c, fasting glucose, blood creatinine, insulin, lipids and creatinine with estimation of GFR were performed along with echocardiography at baseline. The observation period was 56 weeks.

**Results:** We identified 76 composite cardiovascular (CV) outcomes, which included CV death and death from all causes, resuscitated cardiac death, non-fatal/fatal acute myocardial infarction or stroke, and HF hospitalization. Therefore, the entire patient cohort was divided into 2 groups with (*n* = 76) and without (*n* = 77) composite CV outcomes. We found that the concentrations of NT-proBNP were higher in HF patients with T2DM who had a CV composite outcome than in patients without CV composite outcome (*p* = 0.001). In contrast, the relationship was exactly reversed for irisin, as HF and T2DM patients with CV composite outcome had significantly lower irisin levels (*p* = 0.001). Unadjusted multivariate Cox regression analyses showed that LVEF < 40%, LAVI > 39 ml/m^2^, NT-proBNP > 2,250 pmol/ml, and irisin < 6.50 ng/ml were the strongest predictors of CV outcomes in HF patients with T2DM. After adjustment for LVEF, serum levels of NT-proBNP and irisin remained independent predictors of end points. Furthermore, divergence of Kaplan-Meier curves pointed out that patients with NT-proBNP > 2,250 pmol/ml and irisin < 6.50 ng/ml had worse prognosis than those with any other compartment of the bomarkers’ levels.

**Conclusion:** Adding irisin to NT-proBNP significantly improved discriminative value of the whole model. HF patients with T2DM had significantly worse clinical outcomes when showing the constellation NT-proBNP > 2,250 pmol/ml and irisin < 6.50 ng/ml, respectively, in comparison to patients with opposite trends for both biomarkers.

## 1 Introduction

The total number of patients with heart failure (HF) is steadily increasing worldwide, mainly due to HF with preserved ejection fraction (HFpEF), whereas in most developed countries the increase in the prevalence of HF with reduced ejection fraction (HrEF) has stabilized in certain populations (Groenewegen et al., 2020; Virani et al., 2021). This is likely related to the growing burden of cardiovascular (CV) and metabolic risk factors such as hypertension, chronic kidney disease, obesity, type 2 diabetes mellitus (T2DM) and population aging (Bouthoorn et al., 2018). However, regardless of the phenotype of HF, patients with known HF have a similarity in 5-year all-cause mortality rate and unacceptably poor clinical outcomes, including CV death and HF admission (Nichols et al., 2015; Shah et al., 2017). On the other hand, T2DM is strongly associated with a 2- to 4-fold increased risk of HF (Park, 2021) and that intensive management of CV risk factors and T2DM reduces the risk of subsequent all-cause death, CV events and HF hospitalization (Upadhya et al., 2021; Dziopa et al., 2022). Current clinical guidelines for HF generally recommend the use of natriuretic peptides (NPs), including the N-terminal fragment of brain natriuretic pro-peptide (McDonagh et al., 2021; Heidenreich et al., 2022), but the predictive discriminatory potency of NPs varies sufficiently in HFrEF and HFpEF (Arshi et al., 2021) as well as in T2DM and obese patients because of high variability of serum levels of NPs (Pieske et al., 2019).

Irisin is one of the myokines produced by skeletal muscle in response to physical exercise and provides a crosstalk between muscle, myocardium, adipose and bone tissues (Colaianni et al., 2016). Under physiological conditions, irisin appears to be a crucial element in adipose tissue browning, regulating glucose metabolism and influencing energy expenditure (Moreno-Navarrete et al., 2013). Irisin has been found to have a tissue-protective effect on these target organs by promoting angiogenesis, reparation, vascular integrity, suppression of oxidative stress, fibrosis, reduction of cardiomyocyte apoptosis and ischemia/hypoxia-induced injury *via* the PI3k/Akt/mTOR and αV-integrin-induced extracellular signaling-related kinase/STAT pathways (Liao et al., 2019; Yang et al., 2021). Unfortunately, there are contradictory findings regarding the role of irisin in HF patients. For example, there is evidence that serum levels of irisin decrease in chronic HF and increase in acute HF due to acute myocardial infarction (Shen et al., 2017; Abd El-Mottaleb et al., 2019), whereas low levels of irisin have been found in T2DM at risk for HF (Akyuz et al., 2021). Besides, it remains unclear whether irisin could add prognostic information to the elevated NP levels in stable HF patients with T2DM who continue to treat with optimal guideline-based therapy. The aim of this study was to investigate the role of serum levels of irisin in predicting clinical outcome in HF patients with T2DM.

## 2 Methods

### 2.1 Study Design and Cohort Identifications

**FIGURE 1:**
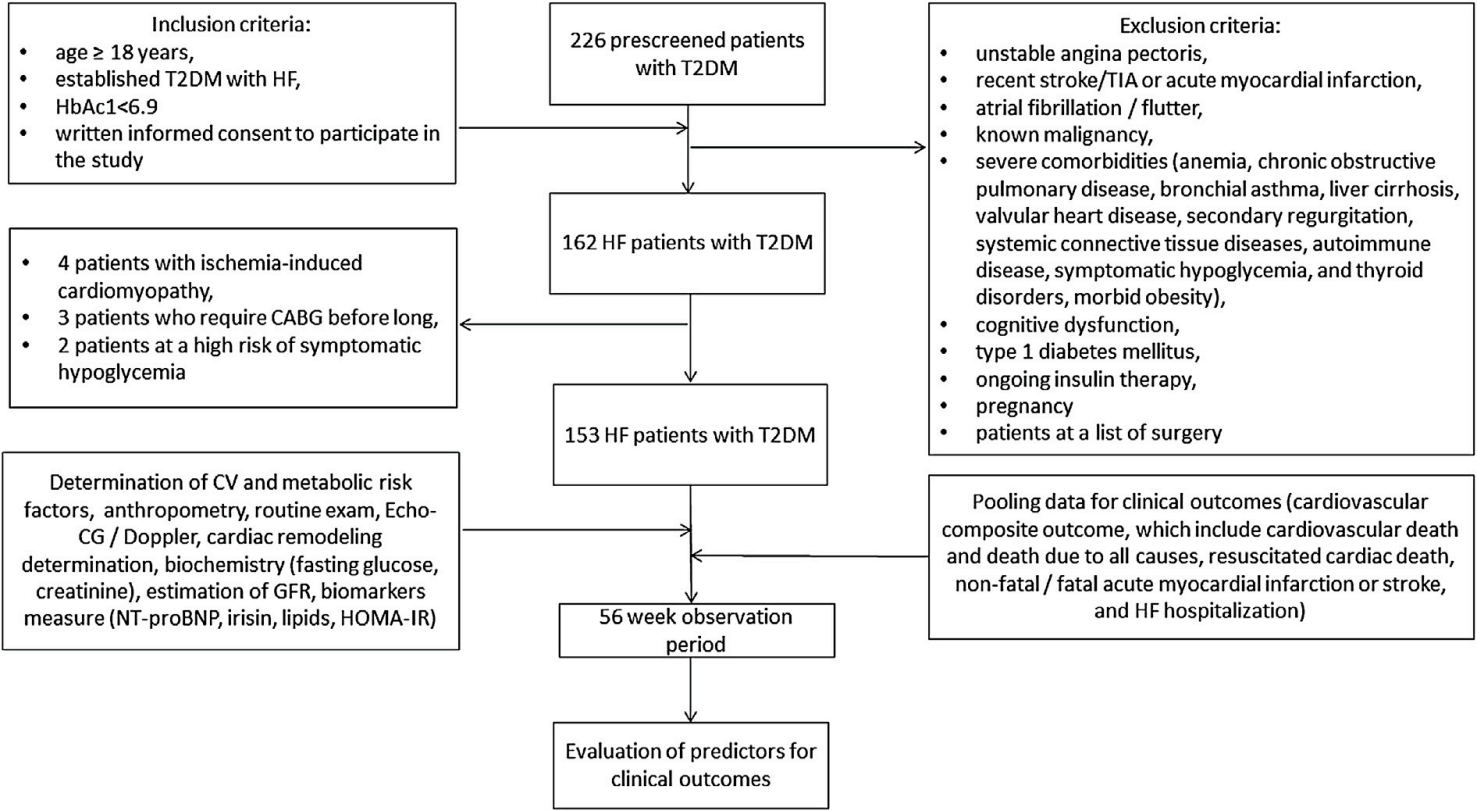
Study design flow chart. Abbreviations: HF, heart failure; T2DM, type 2 diabetes mellitus; CABG, coronary artery bypass grafting; CV, cardiovascular; TIA, transient ischemia attack; Echo-CG, echocardiography; NT-proBNP, N-terminal fragment of pro-natriuretic peptide; HOMA-IR, Homeostasis Model Assessment of Insulin Resistance.

Of 226 prescreened T2DM patients, we enrolled 162 individuals with HF according to the following inclusion criteria: age ≥ 18 years, established T2DM with HF, adequate control of hyperglycemia (HbAc1< 6.9%), written informed consent to participate in the study. Exclusion criteria included unstable angina, recent stroke/transient ischemic attack (TIA), acute myocardial infarction, atrial fibrillation/flutter, known malignancies, severe comorbidities (anemia, chronic obstructive pulmonary disease, bronchial asthma, liver cirrhosis, known valvular heart disease, symptomatic hypoglycemia, morbid obesity, congenital heart disease, systemic connective tissue disease, autoimmune diseases, cognitive dysfunction and thyroid disease), type 1 diabetes mellitus, ongoing insulin therapy and pregnancy. Figure 1 illustrates the flowchart of the study design. Then, we excluded nine subjects who could not be evaluated at follow-up. Finally, 153 HF participants with T2DM aged 41–62 years were prospectively enrolled in the study. All patients were treated at the Vita-Centre private hospital (Zaporozhye, Ukraine) from October 2020 to December 2021.

### 2.2 Ethical Declaration

The study was conducted according to the guidelines of the Declaration of Helsinki and approved by the Institutional Ethics Committee of Zaporozhye Medical Academy of Post-graduating Education (protocol number: 8; date of approval: 10 October 2020). All patients gave voluntary written informed consent to participate in the study.

### 2.3 Data Collection

The demographic data and clinical characteristics including age, gender, etiology of HF, phenotype of HF, comorbidities at admission and final clinical outcomes were recorded for each patient.

### 2.4 Determination of Clinical Outcomes

During the 56-week follow-up period (the median was 52 weeks), we pooled composite CV outcomes that included CV death and death due to all causes, death after resuscitation, non-fatal/fatal acute myocardial infarction or stroke and HF hospitalization. Clinical outcomes were determined by face-to-face contact with patients, their relatives or caregivers (by telephone or/and during hospital or primary care visits) and by reviewing source documents including local databases, discharge reports, physician reports, autopsy reports and other relevant files (ECGs, images, health summaries, responses to inquiries, consultation reports, laboratory reports, etc). Reporting of clinical outcomes was consequently performed by the person in charge (AAB) and the supervisor (IMF) until the end of the study.

#### 2.4.1 Determination of Patients’ Background, Risk Factors and Co-morbidities

We used the guidelines that were valid at the current time of enrollment to determine HF (McDonagh et al., 2021), T2DM (Standards of Medical Care in Diabetes--2017: Summary of Revisions, 2017), dyslipidemia (Catapano et al., 2016) and hypertension (Williams et al., 2018).

### 2.5 Anthropometric Measurements and Clinical Examinations

All patients enrolled in the study underwent general clinical and physical examination. We also measured office blood pressure (BP), heart rate, height, weight, waist circumference, waist-to-hip ratio (WHR) and body mass index (BMI).

#### 2.5.1 B-More Transthoracic Echocardiography and Doppler-Method

B-mode transthoracic echocardiography was carried out at study entry with the diagnostic system “GE Medical Systems” (Freiburg, Germany) by 2.5–3.0 MHz phase probe. Hemodynamic parameters were determined in accordance with current recommendation of the European Association of Cardiovascular Imaging (EACVI) and the American Society of Echocardiography (ASE) (Marwick et al., 2015). Left ventricular (LV) ejection fraction (LVEF) was measured using Simpson method. Left atrial volume and its index (LAVI) along with E/e` ratio and LV myocardial mass index (LVMMI) was calculated accordingly to the current recommendation (Nagueh et al., 2016).

### 2.6 Estimating Glomerular Filtration Rate

CKD-EPI formula was used to estimate glomerular filtration rate (GFR) (Levey et al., 2009).

### 2.7 Insulin Resistance Determination

Homeostatic Assessment Model of Insulin Resistance (HOMA-IR) was provided in order to evaluate the insulin resistance (Matthews et al., 1985).

### 2.8 Circulating Biomarkers

Blood samples were received from all fasting patients at study entry and then collected into barcoded silicone plastic test tubes for further centrifugation (6,000 rpm for 30 min). Afterwards, plasma was pooled to be immediately refrigerated and stored at a temperature −70°С. Laboratory tests, such the levels of lipids including total cholesterol (TC), low-density lipoprotein (LDL-C) cholesterol, high-density lipoprotein (HDL-C) cholesterol and triglycerides (TG), creatinine, insulin, glycosylated hemoglobin (HbA1c) and glucose were performed using Roche P800 analyzer (Basel, Switzerland). Concentrations of serum irisin and NT-proBNP were detected with ELISA kit manufactured by Elabscience (Houston, Texas, United States) using Labline-90 analyzer (Frankenmarkt, Austria) and Elecsys 1010 analyzer (F. Hoffmann-La Roche Diagnostics, Mannheim, Germany), respectively.

### 2.9 Statistical Analyses

We used V. 23 Statistical Packages for Social Sciences (SPSS; IBM, Armonk, New York, United States) software and v. 9 GraphPad Prism (GraphPad Software, San Diego, CA, United States) software for statistical analysis and data presentation. Power Analysis and Sample Size (NCSS Statistical software, Kaysville, Utah, United States) software was determined to calculate the sample size. The following formula (the “Survival” module) was used:

$$n={\left(\frac{Z_{1-a/2}\times \sqrt{p\times \left(1-p\right)}}{\delta }\right)}^{2}$$

Z1-α/2 was 1.96; and the statistical power was 0.90, δ represented allowable error; *p* represented sensitivity or specificity of NT-proBNP, which were determined as 0.78 and 0.88 in previous precious study of HF in predicting adverse outcomes in T2DM (Malachias et al., 2020). Therefore, 153 patients were needed in this study to obtain concise results.

We applied Kolmogorov-Smirnov test to assess normality of continuous variables without transformation when distribution was non-normal. Continuous variables with normally distribution were characterized by mean ± standard deviation (SD), whereas continuous, non-normally distributed variables were specified by median (Me) and interquartile range (IQR). One-way analysis of variance (ANOVA) and *t*-test were used for the comparison between subgroups of the patients. Categorical variables were presented as frequencies and percentages and were compared using Chi-square test (χ^2^) or Fisher’s exact test (F) as appropriate.

Spearman correlation coefficient (r) was estimated to ascertain the relationship between variables. Predictors for outcomes were determined by univariate and multivariate linear regression analysis. Variables that were statistically significant (*p* < 0.1) in the univariate regression or clinically related were incorporated into the multivariate model to identify independent predictors of cumulative CV outcomes. Beta (β) coefficient, odds ratio (OR) and 95% confidence interval (CI) were reported for each predictive model. ROC curves with separate analysis of Youden Index was constructed to assess reliability of the predictive models. DeLong’s test was applied for comparison of AUC of different models. Multivariate Cox regression was performed to detect predictors for cumulative CV outcomes. Predictors of outcomes were confirmed using integrated discrimination indices (IDI) and net-reclassification improvement (NRI). The IDI was equal to the elevation in discrimination slope defined as the mean difference in predicted risks between patients with and without events. The continuous NRI is a nonparametric analog of the IDI and corresponds to twice the difference in probabilities of upward reclassification for events minus for non-events. Kaplan-Meier curves were constructed to represent the distribution of composite clinical CV outcomes and differences between groups were tested with the log-rank test. All tests were 2-tailed, and *p* < 0.05 was considered statistically significant.

## 3 Results

Table 1 illustrates baseline characteristics of the T2DM patients included in the study. The total patient cohort consisted mainly of men aged 41–61 years who had different HF phenotypes, including HFpEF (31.4%), HFmrEF (32%) and HFrEF (36.6%). The proportion of HF patients with NYHA functional class II to III was 67.3%–32.7%. The majority of patients had classic CV risk factors, such as dyslipidemia (86.3%), hypertension (64.1%), smoking (47.7%), and abdominal obesity (45.8%). Median LVEF was 51%. NT-proBNP and irisin levels were 2,388 pmol/ml and 5.35 ng/ml, respectively. The patients enrolled in the study were treated with conventional pharmacotherapy according to current clinical recommendations. We identified 76 composite CV outcomes per protocol, and consequently, the entire patients’ cohort was divided into two groups with (*n* = 76) and without (*n* = 77) composite CV outcomes. Patients in these groups had comparable clinical and demographic characteristics, proportions of HF phenotypes and NYHA classes, CV risk factors and medications. There were no significant differences between groups in BMI, waist circumference, WHR, HOMA-IR, fasting glucose, total cholesterol (TC), low-density lipoproteins (LDL), high-density lipoproteins (HDL) and triglycerides (TG). However, patients who achieved the composite CV outcomes had lower LVEF, higher LVMMI and E/e` than those who did not. Therefore, circulating levels of NT-proBNP were significantly higher and irisin significantly lower in patients with composite CV outcomes than in the group without these outcomes.

**TABLE 1:** Baseline demographic, clinical and hemodynamic characteristics of study population.

Variables	Entire patients’ population (n = 153)	T2DM patients with HF		*p* value
		With composite CV outcome (n = 76)	Without composite CV outcome (n = 77)	
Age, year	52 (41–61)	53(42–62)	52(40–60)	NS
Male, n (%)	99 (64.7)	49 (64.5)	50 (64.9)	NS
HFpEF, n (%)	48 (31.4)	22 (28.9)	26 (33.8)	NS
HFmrEF, n (%)	49 (32.0)	25 (32.9)	24 (31.2)	NS
HFrEF, n (%)	56 (36.6)	29 (38.1)	27 (35.1)	NS
II/III NYHA class, n (%)	103 (67.3)/50 (32.7)	54 (71.0)/22 (29.0)	49 (63.7)/28 (36.4)	NS
Dyslipidemia, n (%)	132 (86.3)	68 (89.5)	64 (83.1)	NS
Hypertension, n (%)	98 (64.1)	53 (69.7)	45 (58.4)	NS
Smoking, n (%)	73 (47.7)	36 (47.4)	37 (48.1)	NS
Abdominal obesity, n (%)	70 (45.8)	33 (43.4)	37 (48.1)	NS
BMI, kg/m^2^	24.8 (22.1–29.6)	24.2 (21.9–26.6)	25.2 (23.2–27.9)	NS
Waist circumference, sm	85.6 ± 2.9	85.1 ± 3.2	85.8 ± 2.4	NS
WHR, units	0.86 ± 0.04	0.84 ± 0.03	0.87 ± 0.05	NS
LVEF, %	51 ± 9	35 ± 4	58 ± 3	0.001
LVMMI, g/m^2^	151 ± 6	157 ± 4	147 ± 4	0.012
LAVI, mL/m^2^	39 ± 8	42 ± 3	35 ± 3	0.052
E/e`, unit	13.9 ± 0.5	15.8 ± 0.3	12.2 ± 0.3	0.001
NT-proBNP, pmol/mL	2,388 (1320–3,410)	2,925 (2,240–3,510)	1087 (770–1315)	0.001
Irisin, ng/mL	5.35 (2.20–6.84)	3.20 (2.10–4.15)	7.50 (5.90–9.63)	0.001
eGFR, mL/min/1.73 m^2^	82 ± 10.0	75 ± 5.4	87 ± 6.7	0.001
HOMA-IR	7.92 ± 3.1	8.14 ± 2.0	7.70 ± 2.4	NS
Fasting glucose, mmol/L	5.84 ± 1.2	5.93 ± 1.3	5.72 ± 1.5	NS
Creatinine, mcmol/L	108.8 ± 12.1	118.3 ± 6.2	102.2 ± 4.3	0.042
HbA1c, %	6.65 ± 0.04	6.67 ± 0.03	6.61 ± 0.03	NS
TC, mmol/L	6.41 ± 0.05	6.42 ± 0.08	6.38 ± 0.08	NS
HDL-C, mmol/L	0.95 ± 0.21	0.95 ± 0.17	0.97 ± 0.19	NS
LDL-C, mmol/L	4.43 ± 0.20	4.46 ± 0.10	4.42 ± 0.11	NS
TG, mmol/L	2.26 ± 0.04	2.27 ± 0.10	2.24 ± 0.12	NS
SGLT2i, n (%)	139 (90.8%)	65 (85.5%)	74 (96.1%)	NS
ACEI/ARB/ARNI, n (%)	153 (100%)	76 (100%)	77 (100%)	NS
Beta-blocker, n (%)	148 (96.7%)	73 (96.0%)	75 (97.4%)	NS
Metformin, n (%)	153 (100%)	76 (100%)	77 (100%)	NS
MRA, n (%)	56 (36.6)	29 (38.1)	27 (35.1)	NS
Loop diuretic, n (%)	137 (89.5%)	76 (100%)	61 (79.2%)	0.046
Statins, n (%)	132 (86.3)	68 (89.5)	64 (83.1)	NS
Antiplatelets, n (%)	153 (100%)	76 (100%)	77 (100%)	NS

Notes: data of variables are given as mean ± SD and as median (interquartile range), *p* values illustrate differences between cohorts with and without CV composite outcome. Variables were compared with ANOVA.

Abbreviations: WHR, Waist-to-hip ratio; BMI, body mass index; SBP, systolic blood pressure; DBP, diastolic blood pressure; LVEF, left ventricular ejection fraction; LVMMI, left ventricle 11 myocardial mass index, left atrial volume index, LAVI; left atrial volume index; E/e`, early dias-tolic blood filling to longitudinal strain ratio; LDL-C, low-density lipoprotein cholesterol; HDL-C, high-density lipoprotein cholesterol; TG, triglycerides; TC, total cholesterol; SGLT2i, sodium-glucose cotransporter-2 inhibitor; ACEI, angiotensin-converting enzyme inhibitor; ARB angioten-sin-II receptor blocker; ARNI, angiotensin receptor neprilysin inhibitor; MRA, mineralocorticoid receptor antagonist; NS, not significant.

### 3.1 Spearman Correlation Between Irisin Levels, Hemodynamics Parameters and Other Biomarkers

We found that irisin levels correlated positively with NT-proBNP levels (r = 0.40, *p* = 0.001), LVEF (r = 0.36; *p* = 0.001) and NYHA class (r = 0.34; *p* = 0.001) and inversely with BMI (r = −0.30, *p* = 0.001), WHR (r = −0.25, *p* = 0.001) and LAVI (r = −0.24, *p* = 0.001). No correlation was detected between circulating irisin levels and age, sex, anthropometric parameters, HOMA, eGFR and concomitant medication use.

### 3.2 ROC Curve Analysis

**FIGURE 2:**
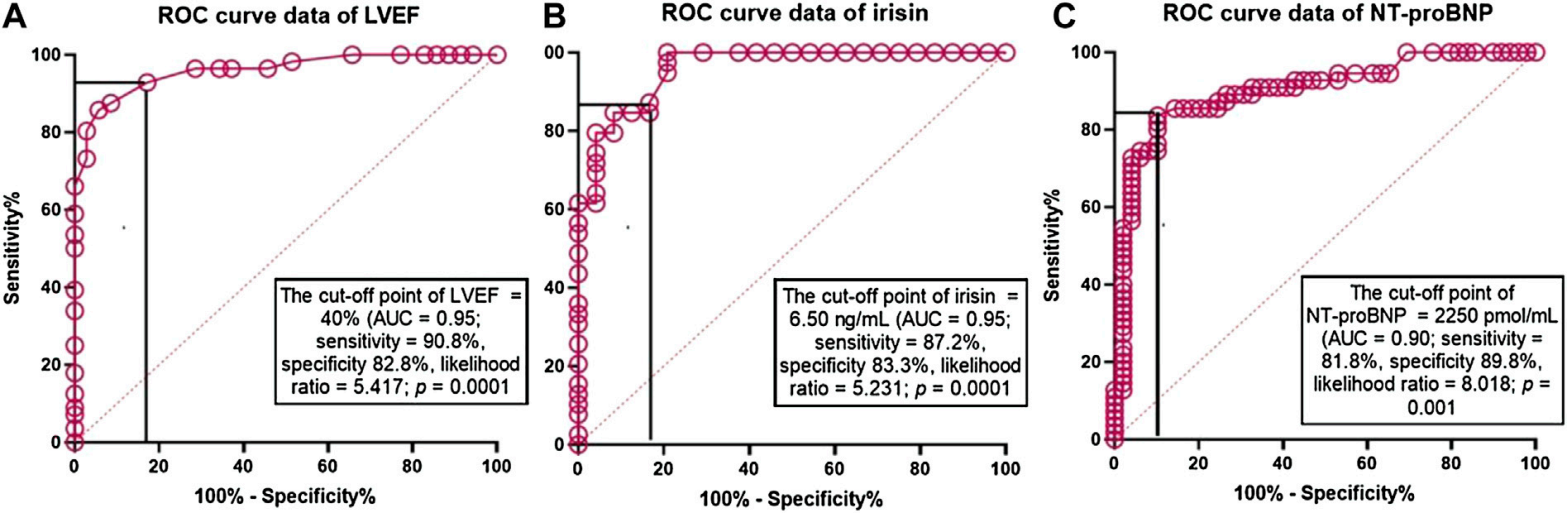
The predictive reliability of LVEF, seru, levels of irisin and NT-proBNP for cumulative CV outcomes: The results of ROC curve analysis. Receiver Operating Characteristic Curves illustrate reliability of predictive models constructed from LVEF, serum levels of irisin and NT-proBNP for cumulative CV outcomes. Abbreviations: AUC, area under curve; NT-proBNP, N-terminal fragment of pro-brain natriuretic peptide.

ROC curve analysis revealed that LVEF < 40% (Figure 2A), serum irisin < 6.50 ng/ml (Figure 2B) and NT-proBNP > 2,250 pmol/ml (Figure 2C) were reliable models for predicting CV composite outcomes in HF patients with T2DM. Sensitivity and specificity of the models were 90.8% and 82.8% for LVEF < 40%, 87.2% and 83.3% for serum irisin levels < 6.50 ng/ml, and 81.8% and 89.8% for NT-proBNP > 2,250 pmol/ml, respectively.

#### 3.2.1 Univariate and Multivariate Linear Regression

Univariate logistic regression showed that composite CV outcomes were predicted by the following variables: LVEF < 40% (OR = 1.07; *p* = 0.016), LVMMI (OR = 1.05; *p* = 0.001), LAVI (OR = 1.04; *p* = 0.001), E/e` (OR = 1.03; *p* = 0.018), serum irisin < 6.5 ng/ml (OR = 1.07; *p* = 0.001) and NT-proBNP > 2,250 pmol/ml (OR = 1.08; *p* = 0.001) (Table 2). Multivariate logistic regression revealed that LVEF (OR = 0.94; *p* = 0.022), serum irisin (OR = 0.89; *p* = 0.001) and NT-proBNP (OR = 1.10; *p* = 0.001) remained independent predictors of CV composite outcomes. Adjusting the regression model to LVEF yielded that only serum irisin < 6.5 ng/ml and NT-proBNP > 2,250 pmol/ml were independent predictors of the dependent variable.

**TABLE 2:** Factors contributing CV composite outcomes in HF patients with T2DM: Results of univariate and multivariate logistic regression analysis.

Variables	Depending variable: CV composite outcomes							
	Univariate logistic regression				Multivariate logistic regression			
	β	OR	95% CІ	Р value	β	OR	95% CІ	Р value
Unadjusted Regression Models								
LVEF<40%	1.32	1.07	1.02–1.12	0.016	2.20	1.09	1.04–1.15	0.001
LVMMI	1.28	1.05	1.01–1.11	0.024	1.22	1.04	1.00–1.90	0.680
LAVI	0.89	1.04	1.00–1.07	0.82	-			
NT-proBNP>2,250 pmol/ml	4.65	1.08	1.04–1.14	0.001	4.15	1.10	1.05–1.16	0.001
Irisin<6.5 ng/ml	4.49	1.07	1.03–1.12	0.001	3.90	1.09	1.03–1.17	0.001
eGFR	0.96	1.02	1.00–1.06	0.058	-			
E/e`	3.20	1.03	1.01–1.04	0.018	2.90	1.03	1.00–1.04	0.058
Regression Models Adjusted to LVEF								
NT-proBNP>2,250 pmol/ml	4.12	1.07	1.03–1.11	0.001	3.90	1.10	1.04–1.17	0.001
Irisin<6.5 ng/ml	4.20	1.05	1.02–1.09	0.001	4.16	1.09	1.04–1.14	0.001

Abbreviations: NT-proBNP, N-terminal fragment of pro-brain natriuretic peptide; eGFR, estimated glomerular filtration rate; LVEF, left ventricular ejection fraction; LVMMI, left ventricle myocardial mass index, left atrial volume index, LAVI; left atrial volume index; E/e`, early diastolic blood filling to longitudinal strain ratio; β, beta-coefficient; SD, standard deviation; OR, odds ratio; CI, 95% confidence interval.

### 3.3 Multivariate Cox Regression Analyses

Unadjusted multivariate Cox regression analyses demonstrated that LVEF < 40%, LAVI > 39 ml/m^2^, NT-proBNP > 2,250 pmol/ml and irisin < 6.50 ng/ml were the strongest predictors for CV composite outcomes in HF patients with T2DM. After adjustment for LVEF, serum levels of NT-proBNP and irisin remained independent predictors of the dependent variable (Table 3).

**TABLE 3:** Multivariate Cox regression analysis of determinant variables affecting CV composite outcomes in HF patients with T2DM.

Variables	Depending variable: CV composite outcomes					
	Unadjusted Cox regression			Adjusted Cox regression		
	OR	95% CI	*p* value	OR	95% CI	*p* value
LVEF<40% vs. ≥ 40%	1.08	1.04–1.13	0.001	Adjustment factor		
LAVI >39 ml/m^2^ vs. ≤ 39 ml/m^2^	1.04	1.01–1.07	0.040	1.02	1.00–1.04	0.80
NT-proBNP >2,250 pmol/ml vs. ≤ 2,250 pg/ml	1.07	1.04–1.11	0.001	1.10	1.06–1.17	0.001
Irisin <6.50 ng/ml vs. ≥ 6.50 ng/ml	1.10	1.04–1.18	0.001	1.10	1.05–1.16	0.001
Model C-index = 0.816						

Abbreviations: LVEF, left ventricular ejection fraction; LAVI, left ventricular volume index.

### 3.4 Comparison of the Predictive Models

Table 4 illustrates the strict similarity of the predictive abilities of model 1 (LVEF < 40%) and other models based on the isolated use of NT-proBNP (model 2) or irisin (model 3), whereas NT-proBNP together with irisin (model 4) significantly improved the predictive value of model 1 and additionally reclassified cases previously classified as event-free.

**TABLE 4:** Statistics for model fit for the prediction of CV composite outcomes

Predictive models	Depended variable: CV composite outcomes					
	AUC		NRI		IDI	
	M (95%CI)	*p* value	M (95%CI)	*p* value	M (95%CI)	*p* value
Model 1 (Based Model: LVEF<40%)	0.83 (0.80–0.85)	-	Reference	-	Reference	
Model 2 (NT-proBNP>2,250 pg/ml) vs. Model 1	0.86 (0.83–0.89)	0.054	0.11	0.44	0.10	0.42
Model 3 (Irisin<6.5 ng/ml) vs. Model 1	0.85 (0.79–0.90)	0.055	0.14	0.18	0.13	0.12
Model 4 (NT-proBNP>2,250 pg/ml + Irisin<6.5 ng/ml) vs. Model 1	0.92 (0.89–0.95)	0.044	0.25	0.04	0.16	0.04

Abbreviations: M, median; CI, confidence interval; AUC, area under curve; ACR, adverse cardiac remodeling; IDI, integrated discrimination indices; NRI, net-reclassification improvement.

#### 3.4.1 Comparisson of the Incidence of Adverse Events for HF Patients With T2DM Depending on the Levels of NT-proBNP and Irisin

**FIGURE 3:**
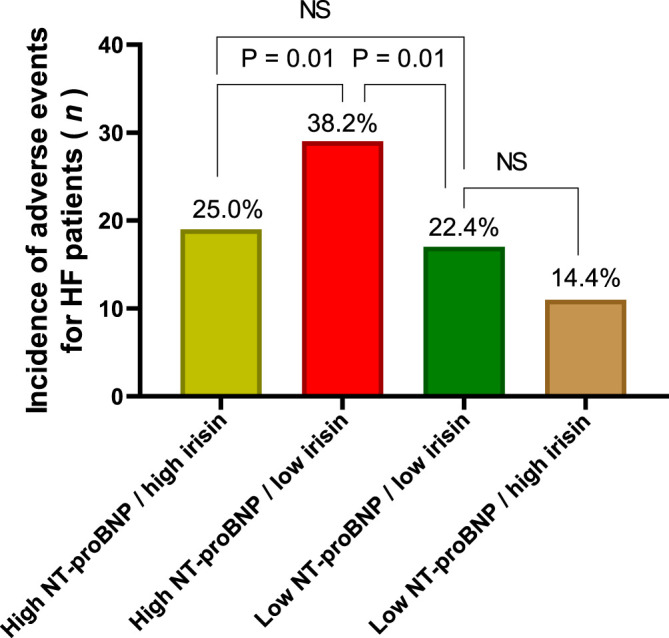
The incidence of CV composite outcomes depending on the compartment of circulating biomarkers. Note: High NT-proBNP means NT-proBNP levels > 2,250 pmol/ml, low NT-proBNP means NT-proBNP levels ≤ 2,250 pmol/ml; high irisin means irisin levels ≥ 6.50 ng/ml; low irisin means the levels of irisin < 6.50 ng/ml.

We compared the incidence of clinical outcomes for HF patients with different levels of the circulating biomarkers. With this aim we constructed four subgroups from the HF patients with clinical end points depending on high/high levels low/low, low/high, high/low, and of NT-proBNP and irisin, respectively (Figure 3). The first subgroup had NT-proBNP levels > 2,250 pmol/ml and irisin < 6.50 ng/ml, the second subgroup had NT-proBNP levels ≤ 2,250 pmol/ml and irisin ≥ 6.50 ng/ml, the third and forth groups of the patients had NT-proBNP levels < 2,250 pmol/ml and irisin < 6.50 ng/ml and NT-proBNP levels > 2,250 pmol/ml and irisin > 6.50 ng/ml, respectively. In fact, HF patients with high/low compartment of the biomarkers demonstrated a significant increase in CV composit clinical outcomes.

### 3.5 Results of Kaplan-Meier Curves

**FIGURE 4:**
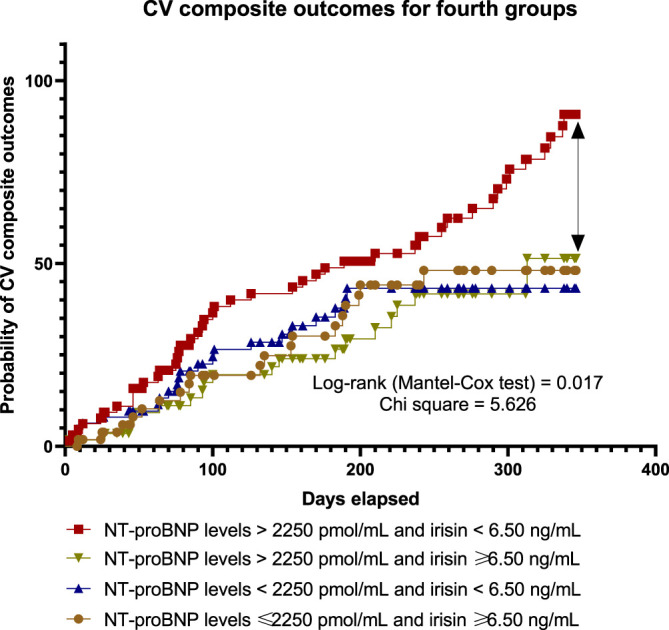
Kaplan-Meier curves illustrate a difference between subgroups in accumulation of pre-specified clinical outcomes depending on the levels of circulating biological markers.

With the aim of comparing the difference in accumulation of clinical outcomes between four subgroups of HF patients with T2DM during 56-week observation period depending on the compartments of circulating biological markers (high/high levels low/low, low/high, high/low, and of NT-proBNP and irisin, respectively) we computed Kaplan-Meier curves (Figure 4). There was a significant difference among the high/low groups when compared others in clinical prognosis. Finally, we found that patients in the group with NT-proBNP > 2,250 pmol/ml and irisin < 6.5 ng/ml had a worse prognosis than other patients with any compartments of the biomarkers’ levels (log-rank test = 0.017).

## 4 Discussion

The results of the study showed that irisin was able to add predictive information to NT-proBNP, thereby improving the discriminatory value of the model applied to HF patients with T2DM. We first found that HF patients with T2DM had significantly worse clinical outcomes when showing the constellation NT-proBNP > 2,250 pmol/ml and irisin < 6.50 ng/ml, respectively, in comparison to patients with opposite trends for both biomarkers. Like other investigators, we did not find a strong correlation between irisin and metabolic parameters, including the HOMA index in T2DM with known HF (Abd El-Mottaleb et al., 2019; Silvestrini et al., 2019), but a weak inverse correlation of the biomarker with BMI, WHR and NT-proBNP was observed. However, we also confirmed that irisin levels are multidirectional to NT-proBNP and are associated with the severity of HF. This supports the hypothesis that irisin acts as an adaptive hemodynamic and metabolic regulator of cardiac function in fluid overload, cardiac remodeling and other aspects of HF (Berezin et al., 2021).

Indeed, over-expression of irisin in cardiomyocytes mediated adaptive autophagy and attenuated cardiomyocyte apoptosis, so that irisin prevented cardiomyocyte loss due to angiotensin II-induced injury and biomechanical stress (Li et al., 2019). Thus, irisin strongly increases cellular proliferation, prevents cardiomyocyte apoptosis and decreases intracellular reactive oxygen species (ROS) levels induced by H_2_O_2_ (Peng et al., 2017). On the other hand, the functional status of HF patients seems to be strongly associated with plasticity of skeletal muscle metabolism, which is simultaneously enhanced by the expression of the peroxisome proliferator-activated receptor-γ (PPARγ) coactivator-1α and the fibronectin type III domain containing 5 (FNDC5) gene, which encodes the production of irisin (Lecker et al., 2012; Tiano et al., 2015). Indeed, low irisin levels may underlie altered CV homeostasis and reduced aerobic performance in HF patients, influencing HF progression and worsening clinical outcomes (Zhang et al., 2021).

Along with it, irisin is produced not only by skeletal muscles but also by thermogenic cells originated from white adipocytes in result of their browning (Arhire et al., 2019). However, physical exertion seems to be a main promotor for inducing expression of PPARγ coactivator-1 and downstream FNDC5, cleavage of which is mediated by irisin (Nesti et al., 2021). Therefore, accumulation of epicardial adipose tissue is considered to be a predictor of poor clinical outcomes in HFpEF, while its role in promoting adverse clinical outcomes in relation to cardiac remodeling and synthesis and secretion of irisin is under scientic discussion (Nesti et al., 2020; Pugliese et al., 2021).

Although irisin was recently found to be a predictor of cardiac cachexia (Sobieszek et al., 2020; Qaisar et al., 2021), its ability to predict the clinical course of CV disease in stable HF patients remained unclear. Hsieh et al. (2018) reported that an increased risk of CV mortality, stroke, HF and revascularization in patients with acute ST-elevation myocardial infarction was mainly documented in those who had the highest circulating irisin levels. In contrast, Matsuo et al. (2015) found that the expression of FNDC5 in skeletal muscle was sufficiently reduced in ischemic cardiomyopathy. Moreover, T2DM was associated with low irisin levels (Akyuz et al., 2021), and a decrease in serum irisin was not related to the occurrence of obesity in these patients (Standards of Medical Care in Diabetes--2017: Summary of Revisions, 2017). Therefore, clinical importance of variable irisin concentrations in HF patients with metabolic comorbidities including T2DM required a clear explanation. (Verma et al., 2022).

In the study, we included stable T2DM patients with NYHA class II/III HF receiving optimal therapy. We figured out that irisin had demonstrated its predictive potency in a similar manner to NT-proBNP and was able to increase the discriminatory ability of this peptide. We hypothesized that in euvolemic or near-euvolemic HF patients with T2DM treated with optimal guideline-recommended therapy, the predictive power of persistent NT-proBNP levels might be far from ideal and that the additional serum levels of irisin could be a powerful prognosticator for long-term prognosis. The results of the study were completely consistent with our previous hypothesis, while direct comparison of discriminatory potencies of both biomarkers provided evidence for their comparability. Future large clinical trials are needed to clarify the role of the combined predictive model in T2DM patients with different HF phenotypes.

## 5 Study Limitations

The study has some limitations, which mainly include the small sample size and the single-center design. Although we calculated the sample size before enrolling patients in the study, the largest number of HF patients with T2DM by far could have allowed us to compare the predictive power of the biomarker models for different HF phenotypes. In fact, we refused this analysis because of the limited number of patients in each HF category. In addition, the single center study design ensured a standard approach to the management of HF and T2DM according to international and local clinical protocols, which is considered to introduce some bias in the recruitment of patients in the study.

The next limitation is the use of atrial fibrillation and atrial flutter as exclusion criteria. We chose to minimize the biologic variability of biomarkers of HF, such as NT-proBNP and irisin, because stable HF patients with the same NYHA class and LVEF with sinus rhythm and atrial fibrillation/flutter have enormous differences in serum levels of these molecules. In routine clinical practice, HF patients with NT-proBNP levels > 2,250 pmol/ml would have a high proportion of atrial fibrillation. Thus, predictive cut-off points for both biomarkers would not be concisely determined. Otherwise, we would need to construct models for HF patients with sinus and non-sinus rhythm separately.

In addition, we did not include patients in whom further surgical procedures, revascularization, etc., was planned. In addition, we did not include individuals with high variability in circulating biomarkers because of impaired clearance, reduced eGFR and morbid obesity. However, we believe that these limitations are not relevant to the interpretation of the data.

## 6 Conclusion

Adding irisin to NT-proBNP significantly improved discriminative value of the whole model. HF patients with T2DM had significantly worse clinical outcomes when showing the constellation NT-proBNP > 2,250 pmol/ml and irisin < 6.50 ng/ml, respectively, in comparison to patients with opposite trends for both biomarkers. These findings may open new approaches to manage HF in T2DM using point-of-care biomarkers models in the future.

## Data Availability

The original contributions presented in the study are included in the article/Supplementary Material, further inquiries can be directed to the corresponding author.

## Abbreviations

ACE, angiotensin-converting enzyme; Akt, protein kinase B; ARB, angiotensin-II receptor blocker; BMI, body mass index; BP, blood pressure; CAD, coronary artery disease; CV, cardiovascular; CVD, cardiovascular disease; ERK 1/2, Extracellular signal-regulated kinase ½; HDL-C, high-density lipoprotein cholesterol; HF, heart failure; HFmrEF, heart failure with mildly reduced ejection fraction; HFpEF, heart failure with preserved ejection fraction; HFrEF, heart failure with reduced ejection fraction; HOMA-IR, homeostasis model assessment insulin resistance; IDI, discrimination indices; LAVI, left atrial volume index; LDL-C, low-density lipoprotein cholesterol; LV, left ventricle; LVEF, left ventricular ejection fraction; MAPK, mitogen-activated protein kinase; mTOR, mammalian target of rapamycin; NRI, net-reclassification improvement; PI3K, phosphatidylinositol 3-kinase; ROS, reactive oxygen species; SGLT2, Sodium-glucose cotransporter-2; STAT, Signal transducer and activator of transcription; T2DM, type 2 diabetes mellitus; TC, total cholesterol; TG, triglycerides.
